# Lectures on the Principles and Practice of Medicine

**Published:** 1884-11

**Authors:** 


LECTURES ON THE PRINCIPLES AND PRACTICE OF MEDICINE. *Delivered in Chicago Medical College, Medical Department of the Northwestern University, by* NATHAN SMITH DAVIS, A.M., M.D., L.L.D., *Dean of the Faculty and Professor of Principles and Practice of Medicine and Clinical Medicine in Chicago Medical College, etc., etc. 8vo., pp. x., 896. Chicago:* JANSEN, McCLURG & CO., 1884.

Davis's Principles and Practice of Medicine is one of a number of good books which have been issued by the American press during the present year. Owing to the prominent position of its author in the medical profession, and community, this book is destined to arouse an unusual degree of interest and provoke acrimonious criticism. Dr. Davis has acted wisely in embodying the views and modes of practice, developed during "fifty years of constant devotion to the study and practice of the healing art," in one volume of ninety-two lectures. The book is thus placed completely out of the pale of censorious criticism. Cavilers are at once disarmed, and the rules of criticism applicable to a systematic treatise on the theory and practice of medicine are unsuited to the review of the book under consideration. Then, too, by the adoption of the style of conversational lectures the book acquires an intensely practical character without losing in theoretical interest. Billroth adopted, with obvious advantage, a strictly similar method in the composition of his justly famous "Fifty Lectures on General Surgical Pathology and Therapy."

In Part First the elementary consideration or principles of medicine are discussed in a clear, distinct, vigorous manner. *Vis medicatrix naturæ* receives appropriate and adequate recognition. The suggestions as to the examinations of the sick are of an eminently happy and practical character. The consideration of individual diseases is confined to Part Second. In the discussion of individual diseases, a concise history of each affection is narrated. Too much praise cannot be bestowed upon this feature of the treatment of the subject. Knowledge of the history of disease is necessary to adequate comprehension of medical science. For a long time this subject has been neglected by systematic American and English authors. This omission has not escaped the attention of German and French writers, whose frequent, pointed allusions in the recent International Medical Congress are known to every one.

The chapters on Etiology and Pathological Anatomy give evidence of critical study of most recent developments. Dr. Davis does not believe in the causative influence of "bacillus tuberculosis," or the comma bacillus. In this conviction he is thoroughly in accord with many, perhaps most, eminent clinicians. Morbid structural changes are sketched with as much attention to detail as the general plan of the book will permit.

The vivid pictures of individual diseases, in the chapters on symptomatology and diagnosis, resemble in force and vigor the faithful delineations of the late Doctor Geo. B. Wood.

Dr. Davis's views of treatment are well considered and rational. He is not skeptical as to the physiological action of drugs, and one's faith in therapeutics must be strengthened by a study of the book. The ninety-second chapter is devoted to the discussion of the therapeutic value of alcoholic liquids in the practice of medicine. The author's convictions upon this subject are so well-known that it is scarcely necessary to make the following quotation: "And whoever will boldly make the trial will find that his patients, of every kind, will make better progress on good air and simple nourishment, without any admixture of alcoholic liquids, than they will with such addition."

Few practitioners will be found willing to accept such a *dictum* as settling a question still *sub judice.*

The incorporation of the metric system into the text of the volume marks an important departure from time-honored precedent. All embarrassment on the part of practitioners, educated to the exclusive use of the apothecaries' system, is obviated by the enclosure of equivalents in brackets.

The prominence given to current American medical literature, and to the consideration of the conditions influencing disease in North America,—while not impairing the symmetrical exposition of medical science, viewed absolutely,—adds vastly to the worth of the book for the American practitioner, and renders it unique in medical literature.

The book is not without faults. Errors in spelling and syntax are numerous. On page 13 the following sentence occurs: "To obviate these objections, and at the same time to add another important element to the comparison, I selected a good sized, healthy dog, and while stunned by a blow on the head, quickly opened the abdomen," etc., etc. From the construction of this sentence it is uncertain whether the sensibility of the operator, or of the dog, was benumbed.

These errors, however, are not vital, and do not influence the real value of the production. A careful revision of the book will eliminate them.

Finally, in recommending the book to students and practitioners, we have no hesitation in placing it among those "few" books, "to be chewed and digested," that is, "to be read wholly, and with diligence and attention." W. W. J.

